# From injury to degeneration: delayed lipid-mediated neurotoxicity in a human astrocyte traumatic brain injury in-a-dish model

**DOI:** 10.1038/s41419-026-09217-8

**Published:** 2026-08-24

**Authors:** Luise Schlotterose, Michel A. Struwe, Axel J. Scheidig, Niek Blomberg, Martin Giera, Kirsten Hattermann, Mootaz M. Salman

**Affiliations:** 1 Department of Physiology, Anatomy, and Genetics, University of Oxford, Oxford, UK; 2 British Heart Foundation (BHF) - UK Dementia Research Institute (UK DRI) Centre for Vascular Dementia Research (CVDR) at the University of Oxford, Oxford, UK; 3 Kavli Institute for NanoScience Discovery, University of Oxford, Oxford, UK; 4 British Heart Foundation (BHF) Oxford Centre of Research Excellence, University of Oxford, Oxford, UK; 5 Department for Structural Biology, Kiel University, Kiel, Germany; 6 Center for Proteomics and Metabolomics, Leiden University Medical Center, Leiden, The Netherlands; 7 Institute of Anatomy, Kiel University, Kiel, Germany

**Keywords:** Cellular neuroscience, Trauma

## Abstract

Traumatic brain injury (TBI) is a major cause of long-term neurological disability, and repeated mild TBI (rmTBI) is increasingly linked to chronic neurodegeneration. Clinical evidence suggests that rmTBI creates a prolonged window of vulnerability to further injury. However, the astrocyte-intrinsic mechanisms that convert repeated mechanical stress into delayed neuronal injury remain poorly defined. In particular, whether rmTBI induces a distinct lipid-mediated neurotoxic programme in human astrocytes is unknown. Using a human astrocyte in vitro platform of rmTBI, we deliver controlled repeated mild injuries to astrocytes. Repeated injury induces a distinctive astrocytic phenotype characterised by cytoskeletal remodelling, reactive oxygen species accumulation, biphasic volume changes, extracellular matrix remodelling and lipid droplet formation. Shotgun lipidomics of astrocyte-conditioned medium reveals selective enrichment of bioactive diacylglycerols (DG) following rmTBI. When applied to human cortical neurons, this conditioned medium impairs neuronal function and morphology, increases apoptosis and activates protein kinase C delta (PKCδ). Pharmacological inhibition of PKC attenuates the neurotoxic effect of rmTBI astrocyte-conditioned medium. These findings support a model in which human astrocytes retain a form of ‘injury memory’ through sustained phenotypic and lipid metabolic remodelling. By implicating the DG-PKCδ axis in delayed neurotoxicity, the study identifies PKC signalling as a tractable target for limiting the long-term consequences of rmTBI.

**Figure:**
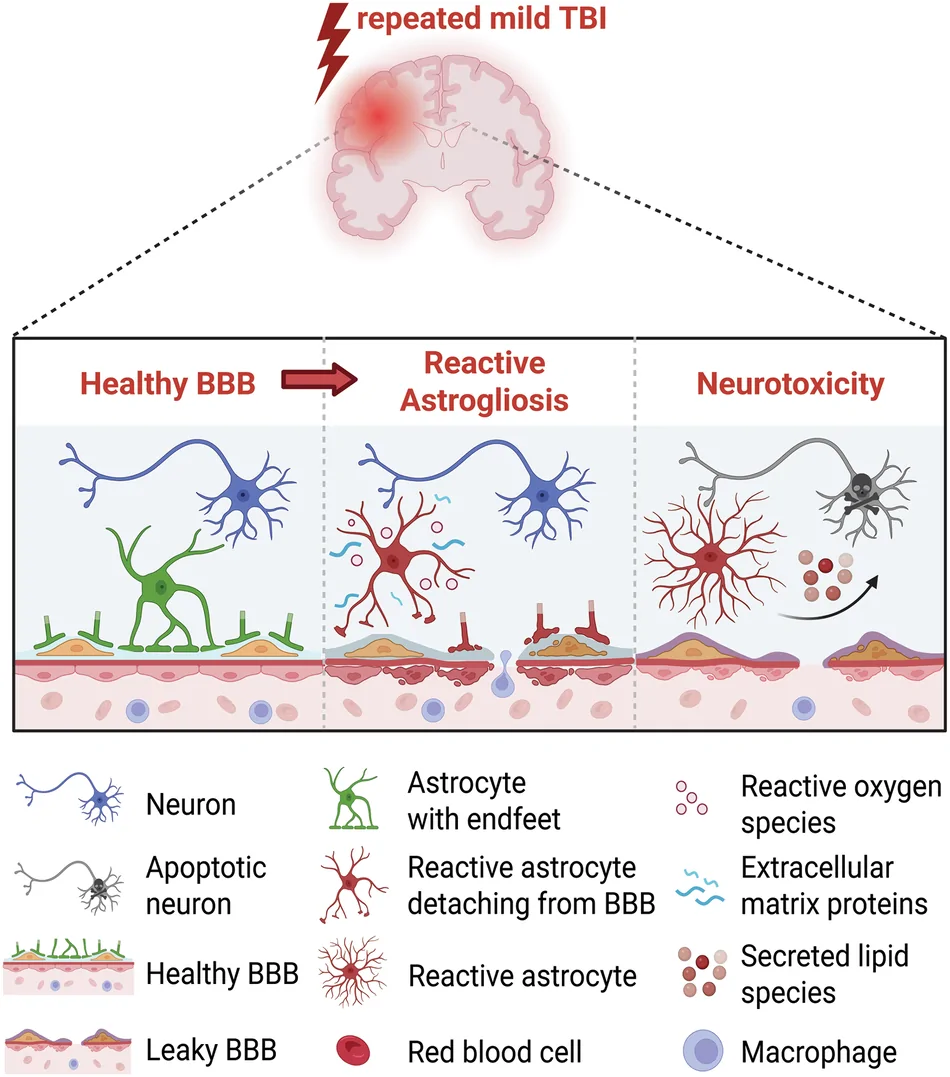
Repeated mild traumatic brain injury induces astrocytic dysfunction, metabolic imbalance and disrupts lipid homeostasis. This leads to the aberrant release of specific diacylglycerol species which activate protein kinase C signalling in neurons and drive delayed neurotoxic cascades.

Repeated mild traumatic brain injury induces astrocytic dysfunction, metabolic imbalance and disrupts lipid homeostasis. This leads to the aberrant release of specific diacylglycerol species which activate protein kinase C signalling in neurons and drive delayed neurotoxic cascades.

## Introduction

Traumatic brain injury (TBI) is a leading cause of neurological disability, affecting approximately 69 million people worldwide each year [1, 2]. Mild TBI (mTBI), including concussion, accounts for the vast majority of cases, but is frequently underdiagnosed, particularly in community, sport and military settings [3–5]. While most patients clinically recover within weeks, approximately 10–20% develop persistent cognitive, emotional or somatic symptoms and epidemiological studies link a history of TBI to increased risk of late-life neurodegenerative disease [6, 7]. Repeated mild TBI (rmTBI) is of particular concern: even when each impact is clinically ‘mild’, clustered injuries are associated with chronic traumatic encephalopathy (CTE) and other progressive tauopathies [8–10].

Experimental work has shown that even a single mTBI triggers a characteristic neurometabolic crisis, with reductions in ATP, *N*-acetylaspartate and acetyl-CoA, impaired mitochondrial function and altered glucose handling [11–13]. This bioenergetic imbalance defines a temporal ‘window of vulnerability’, during which a subsequent insult leads to disproportionately greater histological and functional damage, approximating that of a severe TBI [14, 15]. However, most mechanistic work has focused on neurons, axons and synapses and much less is known about how astrocytes, as central regulators of brain metabolism and homeostasis, convert and propagate the consequences of rmTBI.

Astrocytes are a major class of glial cells in the central nervous system (CNS); strategically positioned at the interface of synapses, vasculature and extracellular space [16]. They provide metabolic support, regulate inflammatory responses and respond rapidly to brain injury with changes in gene expression, morphology and secretory profile [17–19]. Astrocytes also regulate water flux through aquaporin-4 (AQP4) at perivascular endfeet [20] and contribute to glymphatic clearance of metabolic waste [21, 22]. In vivo and ex vivo models of TBI show that reactive astrocytes can be both protective and deleterious, supporting tissue repair in some contexts while driving excitotoxicity, inflammation and blood-brain barrier (BBB) dysfunction in others [23–25]. Yet the cell-intrinsic mechanisms by which astrocytes integrate repeated mechanical insults and transition from an adaptive to a neurotoxic state remain insufficiently defined.

A growing body of evidence points to lipid signalling and lipid damage as critical yet incompletely understood mediators of TBI pathology [26]. Mechanical injury disrupts membrane architecture, activates phospholipases and generates bioactive lipids [27–29]. In parallel, astrocytes accumulate lipid droplets and remodel lipid metabolism under inflammatory and oxidative stress, a response that can be neuroprotective by buffering peroxidised lipids, but also potentially neurotoxic through the release of lipid mediators [30, 31]. Despite this, the causal links between repeated mechanical insults, astrocyte lipid remodelling and delayed neuronal injury remain less defined and the lipid component of this secretome after rmTBI-like stimulation is poorly characterised.

Current experimental models only partially address this gap. In vivo rmTBI models capture important biomechanical features but are heterogeneous and difficult to control at the level of individual cell types and they often require large animal numbers and complex behavioural endpoints [32–34]. Only a few in vitro platforms have been designed to systematically analyse repeated mild insults under controlled conditions, particularly from the perspective of astrocyte-intrinsic responses and lipid-mediated signalling. As a result, mechanistic insight into how astrocytes retain a memory of prior mechanical stress, how their phenotype changes after rmTBI-like stimulation and which lipid species and pathways may act as drivers of secondary neuronal damage is lacking.

Here, we address this gap by implementing and further refining a modular, scalable in vitro TBI model that delivers precisely controlled repeated mild impact injuries to astrocytes, mimicking clinically relevant patterns of rmTBI while retaining experimental tractability [35]. By combining quantitative imaging, biochemical analyses and functional assays on neurons exposed to astrocyte-conditioned medium, we test the hypothesis that repeated mild mechanical injury induces a distinct astrocyte phenotype that is sufficient to drive delayed neurotoxicity. Together, this approach aims to link rmTBI to astrocyte lipid reprogramming and neuron-damaging outputs, providing a mechanistic framework for targeting glial lipid metabolism in the prevention or treatment of long-term consequences of repeated head injuries.

## Materials and methods

### Cell culture

The human foetal astrocyte cell line SVGA was kindly provided by the group of Christine Hanssen Rinaldo, the University Hospital of North Norway [36], with the permission of W.J. Atwood [37]. The cells were grown in Dulbecco’s modified Eagle’s medium (DMEM, #41965, Thermo Fisher Scientific, Germany) supplemented with 10% foetal bovine serum (FBS, #P30-3306, PAN-Biotech GmbH, Germany), 1% penicillin–streptomycin (#15140122, Thermo Fisher Scientific) and 2 mM additional l-glutamine (#56-85-9, Carl Roth, Germany) and incubated at 5% CO_2_/ 37 °C. Astrocytes were seeded at a density of 2000 cells/cm^2^. SVGA cells were used in passages <72. The cells were routinely checked for mycoplasma contamination using a mycoplasma-specific PCR (#11-1100, Venor®GeM Classic; Minerva Biolabs®, Germany).

Primary human foetal astrocytes, isolated from the cerebral cortex, were purchased from ScienCell (HA, #1800, ScienCell, USA) and cultured in Corning® BioCoat® Collagen I cell culture flask (#354485, Corning, USA) in astrocyte medium (AM, #1801, ScienCell). Astrocytes were used in passages < 5. For experiments, astrocytes were seeded at a density of 5000 cells/cm^2^ on plates coated for at least 1 h with 2 µg/cm^2^ poly-l-lysine (PLL, #P4707, Sigma-Aldrich/Merck, Germany) after washing with phosphate-buffered saline (PBS). Primary human foetal neurons, isolated from human cortex (HN, #1520, Sciencell) were cultured in neuronal medium (NM, #1521, Sciencell). After thawing, the neurons were plated directly at a density of 20,000 cells/cm^2^ on plates coated with 2 µg/cm^2^ PLL (#P4707, Sigma-Aldrich/Merck) overnight, washed with PBS, and double-coated with laminin mouse protein (#23017015, Thermo Fisher Scientific). Primary cells were incubated at 5% CO_2_/ 37 °C. All donors are described as normal.

### TBI model

**Fig. 1:**
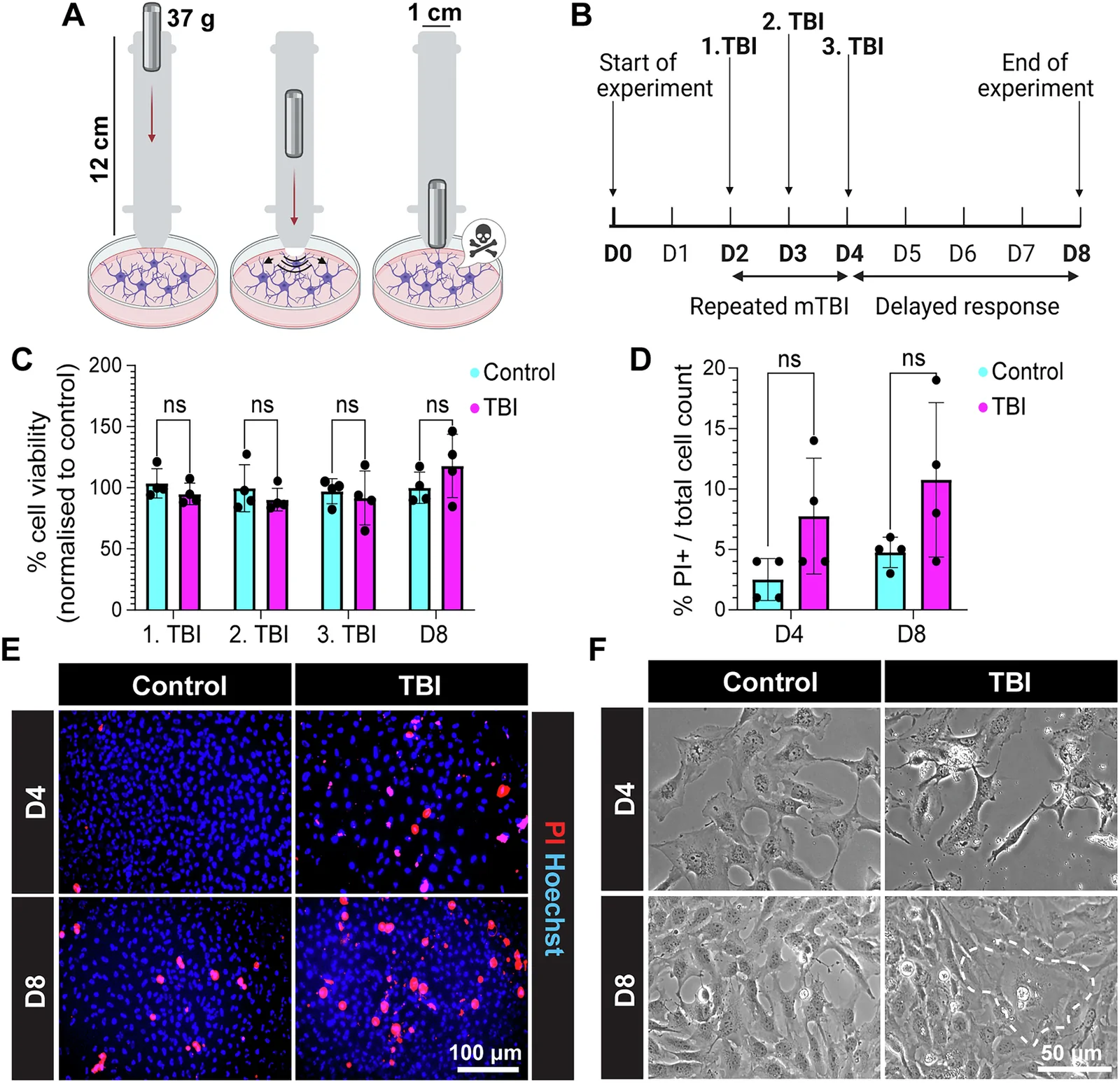
Establishing a modular and scalable in vitro platform that recapitulates repeated mild mechanical injury while preserving astrocyte viability. **A** Schematic of an injury model on human astrocytes. **B** Timeline of experiments. **C** Comparison of cell viability between injured astrocytes and controls shows no significant difference. **D** Cell death staining (PI) confirms that there is no significant increase in cell death following repeated mild TBI. **E** Representative images of dead cell staining: PI/dead cells=red; the cell nuclei were visualized with Hoechst. **F** Representative brightfield images of astrocytes at different time points. TBI samples on day 8 display cell swelling as indicated by the dotted line. **C**–**F** *n* = 4 independent cultures from passaged cells, unpaired two-tailed Student’s t-test, corrected for multiple comparisons using the Holm-Šídák method, *p < 0.05, **p < 0.01, ***p < 0.001, ****p < 0.0001. The bar graphs show mean ± SD.

A mild TBI-like injury was induced as described previously [35]. In brief, the TBI device is a 12 cm high hollow tube 3D printed out of biocompatible resin. The metal weight used was a 37 g metal cylinder with a length of 6 cm and a diameter of 1 cm. A commercially available elastic with 4 cm diameter was attached to the tube and used to ensure the weight was always dropped from the same distance. To injure the cells, the metal weight compressed air within the tube, forming an air bubble in the culture medium. When the weight reached the end of the tube, the air bubble collapsed and released cavitation forces (Fig. 1A). A repeated mild injury was achieved by performing the injury once per day for three consecutive days.

### Drug treatment

1,2-Dilauroyl-sn-glycerol (DG(12:0/12:0/0:0), #15076, Cayman Chemical, USA), 1,3-Dilauroyl-glycerol (DG(12:0/0:0/12:0), #26975, Cayman Chemical) and the ATP-competitive PKC inhibitors GF109203X (#AB120844, Abcam, UK) and Ro-31-8425 (#557514, Sigma-Aldrich/Merck) were dissolved in extra-dry dimethyl sulfoxide (DMSO, #348441000, Thermo Fisher Scientific). DGs were used at a final concentration of 250 ng/ml. GF109203X was used at a final concentration of 0.05 µM and Ro-31-8425 at 0.5 µM.

### Cell viability assays

Cell viability was monitored using a resazurin-based alamarBlue™ Dye (#Y00-100, Thermo Fisher Scientific) for readouts at multiple time points and the CellTiter-Glo® 2.0 Cell Viability Assay (#G9241, Promega, USA), which quantifies ATP, for endpoint assays according to the manufacturer’s instructions. Fluorescence and luminescence intensities were measured in technical duplicates with the CLARIOstar Plus microplate reader (BMG LABTECH, Germany) and normalized to blank wells.

### Cell death and apoptosis assay

Propidium iodide (PI, #P4170, Sigma-Aldrich/Merck) was used to stain for dead cells. Cells were incubated with 40 µg/ml PI and 1 µg/ml Hoechst 33342 (#62249, Thermo Fisher Scientific) to visualise cell nuclei for 30 min at 37 °C. Subsequently 3 areas per sample were visualised with the Keyence BZx800 Fluorescence Microscope (KEYENCE GmbH, Germany).

Apoptosis was analysed using the Caspase-Glo^®^ 3/7 Assay System (#G8090, Promega) in technical duplicates according to the manufacturer’s instructions. The plates were read using a CLARIOstar Plus microplate reader (BMG LABTECH) and normalised to blank wells.

### Reactive oxygen species

Hydrogen peroxide (H₂O₂), a reactive oxygen species, was detected using the ROS-Glo™ H_2_O_2_ Assay (#G8820, Promega) according to manufacturer’s instructions. Samples were measured in technical triplicates using a CLARIOstar Plus microplate reader (BMG LABTECH) and normalised to blank wells.

### Enzyme-linked immunosorbent assays (ELISA)

Cytokine levels were measured in technical duplicates using the IL-1 beta Human ELISA Kit (#KHC0011, Thermo Fisher Scientific), IL-6 Human ELISA Kit (#KHC0061, Thermo Fisher Scientific) and TNF alpha Human ELISA Kit (#KHC3011, Thermo Fisher Scientific) according to the manufacturer’s instructions. Protein content of each sample was determined using the Pierce™ BCA Protein Assay (#23225, Thermo Fisher Scientific) following the manufactures protocol. Plates were read using the CLARIOstar Plus microplate reader (BMG LABTECH), normalised to blank samples and ELISA samples to blanks and protein content.

### Real-time calcium flux measurements

To determine real-time Ca^2+^-transients BMG LABTECH application note AN253 was followed. In brief, cells were incubated in Hank’s Buffer (HBSS) pH 7.4 containing Fluo-4-AM (2 µM, #F14201, Thermo Fisher Scientific), Probenecid (2 mM, #P8761, Sigma-Aldrich/Merck) and Pluronic F-127 (1 mM, #P3000MP, Thermo Fisher Scientific) for 30 min at 37 °C. Once dye loading was observed by fluorescence microscopy, the staining solution was removed and cells were washed with HBSS containing Probenecid (2 mM). After the final wash, cells were allowed to recover for 10 min in fresh HBSS containing Probenecid at 37 °C. Samples were measured in technical triplicates using the CLARIOstar Plus microplate reader (BMG LABTECH) at 37 °C and normalised to blank samples. Fluorescent intensity was read every 0.01 sec for 10 sec per well. An event was defined as ‘signal start’ >2 x SD.

### Quantitative-PCR

The cells were harvested and homogenized using the TRI Reagent® (#93289, Sigma-Aldrich/Merck) and total RNA was isolated following the manufacturer’s protocol. Genomic DNA was digested using RNase-free DNase (1 U/µL) and cDNA was synthesized with the Thermo Scientific RevertAid RT Kit (#K1691, Thermo Fisher Scientific). TaqMan primers and probes (Thermo Fisher Scientific) and HOT FIREPol^®^ Probe Universal qPCR Mix (#08-17-00001, Solis BioDyne, Estonia) were utilized to analyse samples with the ABI PRISM 7500 Sequence Detection System (Applied Biosystems, USA). Analysed genes were GAPDH (Hs99999905_m1), FN1 (Hs00277509_m1), and BGN (Hs00959143_m1).

The cycle threshold (CT) values were determined using the instrument software and ΔCT values were calculated as ΔCT = CT[gene of interest]—CT[GAPDH]. For treatment-induced mRNA regulation, relative expression was calculated using the 2^−ΔΔCT^ method, where ΔΔCT = ΔCT[stimulus] − ΔCT[control]. Thus, fold change = 2−(ΔCT[stimulus]−ΔCT[control]).

### Immunocytochemistry and fluorescent staining

Cells were cultured on glass coverslips. Initially, cells were briefly rinsed with PBS and fixed for 10 min in 4% Paraformaldehyde (PFA) at room temperature. Immunocytochemistry was carried out after permeabilization with 0.1% Triton X-100 (#X100, Sigma-Aldrich/Merck) in PBS for 10 min at room temperature and blocking for 1 h in bovine serum albumin (BSA, 0.5%) and glycine (0.5%) in PBS. Incubation with primary antibodies was performed in blocking solution at 4 °C overnight. The following primary antibodies were used: anti-galectin-3 (goat, 1:100, #AF1154, Bio-Techne GmbH, Germany), anti-Ki67 (mouse, 1:200, #M7240, Dako, USA), anti-AQP4 (rabbit, 1:200, #ab128906, Abcam), anti-MAP2 (chicken, 1:500, #AB92434, Abcam), anti-vimentin (rabbit, 1:500, #ab92547, Abcam) and anti-GFAP (mouse, 1:500, #837204, BioLegend, USA), CoraLite® Plus 555-conjugated anti-TOM20 (rabbit, 1:500, #CL555-80501, Proteintech, UK), Alexa Fluor® 647 anti-PKCδ (rabbit, 1:500, #ab206283, Abcam), ActinRed™ 555 ReadyProbes™ Reagent (2 drops/ml, #R37112, Thermo Fisher Scientific). Afterward, when applicable, samples were incubated with Alexa Fluor 488- or 647-labeled secondary antibodies against the respective host species (donkey IgG, 1:1000, Thermo Fisher Scientific) at room temperature for 2 h and their nuclei were counterstained with 4′,6-diamidino-2-phenylindole (DAPI, #D9542, Sigma-Aldrich/Merck) or Hoechst 33342 (#62249, Thermo Fisher Scientific). The coverslips were embedded with FluroSave Reagent (#345789, Sigma-Aldrich/Merck). For the secondary antibody controls, the primary antibodies were omitted (Fig. 1, Supporting Information). Imaging was carried out using the Keyence BZ-800 fluorescence microscope (Keyence GmbH), EVOS™ M5000 Imaging System (Thermo Fisher Scientific) or Opera Phenix confocal microscope (Revvity, USA). Samples for super-resolution microscopy were prepared in the same manner but without blocking, followed by a 10 min incubation at room temperature with Nile Red (10 µg/ml, #19123, Sigma-Aldrich/Merck) and imaged using a Zeiss LSM 980 Airyscan 2 (Carl Zeiss Microscopy GmbH, Germany).

For extracellular matrix (ECM) staining, samples were briefly rinsed with PBS and fixed for 10 min in 4% PFA at room temperature. The labelling was performed with biotinylated Wisteria floribunda lectin (WFL) (1:100, #B-1355, Vector Laboratories, USA) at room temperature for 1 h followed by incubation with Cy3-conjugated Egg-white Avidin (1:200, #003-160-083, Jackson ImmunoResearch, UK) at 37 °C for 1 h and nuclei were counterstained with DAPI (#D9542, Sigma-Aldrich/Merck). Slides were embedded with Shandon Immu-Mount™ (#FIS9990402, Thermo Fisher Scientific). For secondary labelling controls, primary labelling was omitted (Fig. 1, Supporting Information). Imaging was carried out using the Keyence BZx800 Fluorescence Microscope (KEYENCE GmbH).

In all experiments for each sample > 2 areas were analysed using *ImageJ* (Version 1.54p), National Institutes of Health, USA (RRID: SCR_003070) [38] or *Imaris* Software (Version 10.2.0), Oxford Instruments, UK (RRID:SCR_007370).

### Live microscopy cell swelling assay

Changes in astrocyte cell volume after injury were assessed by visualising the cell bodies with Calcein-AM and live-cell imaging to avoid any changes in cell volume due to the effects of fixation. Briefly, cells were cultured on coverslips and after treatment, the medium was aspirated and exchanged with Calcein-AM staining solution, freshly prepared from 1.5 mL PBS, 1 µL Calcein-AM (1 µg/µl, #C1430, Thermo Fisher Scientific), 2 drops NucBlue^TM^ LiveReadyProbes^TM^ Reagent (#R37605, Thermo Fisher Scientific). Cells were incubated at 37 °C for 20 min and washed with PBS. The coverslip was carefully placed in a live-cell imaging chamber and tightly sealed before covering with FluoroBrite^TM^ DMEM (#A1896701, Thermo Fisher Scientific). Per sample, 3 areas were imaged using the Leica Thunder Imager (Leica Microsystems, Germany) with environmental control (5% CO_2_/ 37 °C) and processed with the small volume computational clearing. Cell volume was quantified with *Imaris* Software (Version 10.2.0), Oxford Instruments (RRID:SCR_007370).

### Transmission electron microscopy

To visualise the functional ultrastructure of astrocytes, cells were fixed in 3% glutaraldehyde in PBS for 30 min. This was followed by post-fixation in 2% osmium tetroxide for two consecutive 5 min incubations and dehydration through a graded ethanol series. Samples were then embedded in a 1:1 mixture of Araldite and ethanol for 60 min at room temperature, polymerized at 40 °C for two days and subsequently cured at 65 °C for one day. The resulting araldite blocks were trimmed for ultrathin sectioning and sections (40–50 nm) were cut using a Leica UC7 ultramicrotome equipped with a diamond knife (Diatom, USA). Sections were contrasted with uranyl acetate for 15 min and lead citrate for 7 min. Ultrastructural analysis was performed using a transmission electron microscope (JEOL JEM-1400Plus) coupled to a digital imaging system (TVIPS TemCam-F416).

### Seahorse real-time metabolic analysis

The oxygen consumption rate (OCR) and the extracellular acidification rate (ECAR) were measured via Seahorse real-time cell metabolic analysis. Cells were cultured in a Seahorse XFe 96 cell culture plate (#103794-100, Agilent, USA) in 30 technical replicates per treatment group. The outermost wells were kept as a blank to normalise during the analysis. One day prior to analysis, the XFe 96 FluxPak (#102416-000, Agilent) was rehydrated with 200 µL of the XF calibrant solution (#100840-000, Agilent) and kept in an incubator without CO_2_ at 37 °C overnight. The following day, cells were washed twice with assay medium, consisting of XF base medium (#103575-100, Agilent) 10 mM D-glucose (#G7021, Sigma-Aldrich), 1 mM sodium pyruvate (#P5280, Sigma-Aldrich) and 2mM l-glutamine (#25030024, Thermo Fisher Scientific). Cells were then topped up with 175 µL of assay medium and incubated without CO_2_ at 37 °C for 1 h. To load the XFe96 FluxPak the following drugs were prepared in assay medium: 1 µM Oligomycin (#O4876, Sigma-Aldrich), 1 µM Carbonyl cyanide-4 (trifluoromethoxy) phenylhydrazone (FCCP, #C2920, Sigma-Aldrich), 0.5 µM Rotenone/Antimycin A (R/A, #R8875, Sigma-Aldrich) and 50 mM 2-Deoxy-Glucose (2-DG #D8375, Sigma-Aldrich). The XFe96 FluxPak was loaded with the drugs in the respective ports and placed into the Seahorse XFe96 Extracellular Flux Analyzer (Agilent). After the pH and O_2_ had been calibrated, the sample XFe 96 cell culture plate was placed into the machine. Once the run was complete, the cells were dry-frozen for subsequent protein quantification with the Pierce™ BCA Protein Assay (#23225, Thermo Fisher Scientific) following the manufacturer's protocol. The OCR and ECAR measurements were normalised to blank controls and protein concentration.

### Clusterin/APOJ analysis

Conditioned medium was subjected to size-exclusion chromatography (SEC) and Clusterin/apolipoprotein J (APOJ) was quantified in the separated fractions using an enzyme-linked immunosorbent assay (ELISA). A Superose 6 Increase 10/300 GL column (#29091596, Cytiva, Uppsala, Sweden) was connected to an ÄKTA Pure 25 M medium-pressure chromatography system (Cytiva), equilibrated in PBS and operated at a flow rate of 0.75 mL × min^-1^. 200 µl of each sample was injected through a capillary loop and fractions of 1.0 mL were collected, beginning after one void volume ( ≈ 0.3 column volumes, 7.068 mL) had passed. Bovine serum albumin was quantified through the area of the characteristic double peak in the UV chromatogram (detection wavelength 280 nm) in the *UNICORN* 7.3 software assuming an extinction coefficient of 0.690 L^–1^ × g. Fractions 6–12 were analysed for their APOJ content using the Human Clusterin ELISA Kit (#EHCLU, Thermo Fisher Scientific) in technical duplicates. APOJ concentrations were normalised to the amount of albumin detected, to compensate for possible variability in the injected sample volume. Absence of APOJ in the culture medium was confirmed by running a blank sample of non-conditioned medium.

### Lipidomics

Shotgun lipidomics analysis across 17 different lipid classes was achieved through differential mobility spectroscopy coupled with tandem mass spectrometry operated with the shotgun lipidomics assistant software (DMS-SLA platform) [39, 40], enabling broad, class-wide profiling of the conditioned medium lipidome. The DMS-SLA platform uses differential mobility spectrometry to separate ions according to their differential mobility in alternating electric fields prior to mass spectrometric analysis. In brief, 10 mL of conditioned astrocyte medium was dried using a SpeedVac and reconstituted in 200 µL LC-MS water before being spiked with an internal standard (IS) mix, covering 74 deuterated lipid species (UltimateSPLASH™ ONE in combination with oleic acid-d9 (dFA 18:1), C13-dihydroceramide-d7 (d18:0-d7/13:0), C15-glucosyl(β)ceramide-d7 (d18:1-d7/15:0), C15-lactosyl(β)ceramide-d7 (d18:1-d7/15:0) and 15:0-18:1-d7-PA) across 17 different lipid classes (with known concentration for each of them). Lipids were extracted using a methyl tert-butyl ether-based protocol. Each lipid species was quantified by the SLA software based on its average intensity resulting from 20 scans, divided by the average intensity of the most structurally similar IS multiplied by its corresponding concentration. A detailed preparation procedure and the DMS-SLA setting can be found elsewhere [41]. Similarly, water blanks and K562 cell aliquots, as quality controls to monitor performance, were prepared using either LC-MS grade water or 1 × 10^6^ cell aliquots.

### Statistical analysis

All results are presented as mean values ± standard deviation (SD) and the number of biological replicates is stated in the respective figure legends, with a minimum of *n* ≥ 3. No samples were excluded from the analysis and no pre-established inclusion or exclusion criteria were applied. Statistical analysis was performed using *GraphPad Prism* (Version 10.6.1), GraphPad Software, USA (RRID: SCR_002798). Comparisons between two groups were performed using an unpaired, two-tailed Student’s *t* test, with Holm-Šídák correction for multiple comparisons where applicable. Comparisons among more than two groups at a single time point were performed using one-way analysis of variance (ANOVA), followed by Tukey’s post hoc test. Comparisons involving the effects of treatment and time were performed using two-way ANOVA, followed by Tukey’s post hoc test for multiple comparisons. The *p*-values *p < 0.05, **p < 0.01, ***p < 0.001 and ****p < 0.0001 were considered statistically significant.

## Results

### A modular and scalable in vitro platform models repeated mild mechanical injury to human astrocytes

Initially, the repeated mild injury model was optimised and validated in immortalised human astrocytes. As previously described, cells were subjected to injury induced by cavitation forces (Fig. 1A) [35]. To verify the astrocytic response, astrocytes were seeded and allowed to settle for 48 h. Cells were then injured once daily for three consecutive days. Following the final injury (day 4), astrocytes were cultured for an additional four days to capture delayed responses (day 8) (Fig. 1B). The acute response to repeated injury was analysed on day 4, 1 h after the last injury.

To ensure the induction of a mild injury, cell viability was assessed after each individual injury and again on day 8 (Fig. 1C). Compared with untreated controls, cell viability did not change significantly at any time point. This was further supported by propidium iodide (PI) staining, which showed no significant increase in cell death on either day 4 or day 8 (Fig. 1D, E). In addition, brightfield imaging revealed injury-associated changes in cell morphology, highlighted by the white dotted line (Fig. 1F).

Therefore, the data indicate that repeated mild injury in our in vitro TBI model does not result in astrocytic cell death, and the injury caused by the cavitation device can be considered ‘mild’.

### Repeated mild impact induces a distinctive reactive astrocyte phenotype

Multiple astrocytic responses to injury in vivo have been described in the literature. To assess the applicability of our in vitro repeated mild TBI model, we examined several previously reported astrocytic injury responses.

**Fig. 2:**
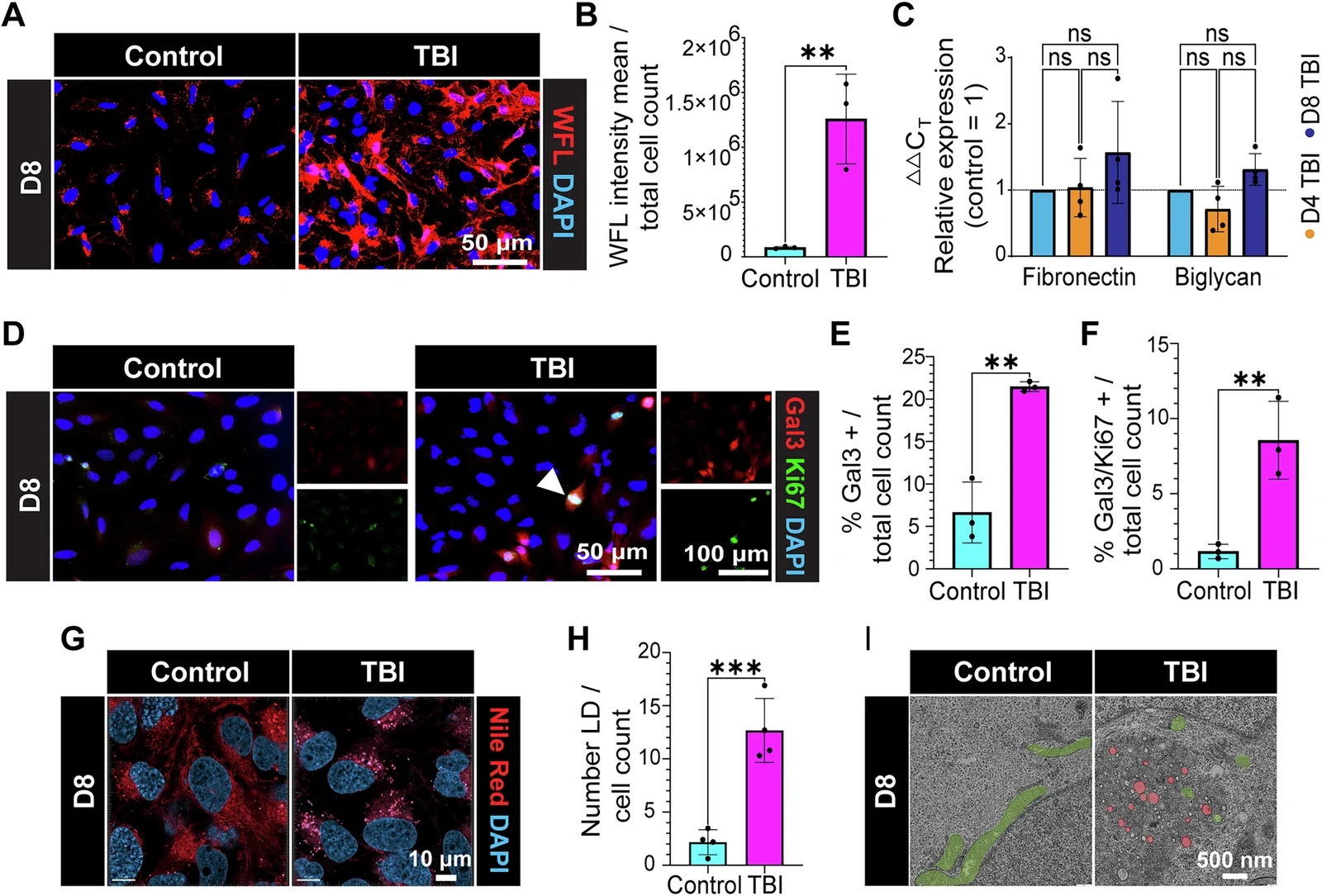
Repeated mild injury induces a distinctive astrocyte phenotype and lipid droplet accumulation. **A** Representative images of immunofluorescence for extracellular matrix proteins (WFL=red) display an increased production on day 8. **B** Quantification of WFL immunofluorescence. **C** Transcript levels analysed by qPCR show an upregulation of ECM remodelling associated genes (Fibronectin, Biglycan) towards day 8. **D** Representative images of immunofluorescence for Galectin-3 (red) and Ki67 (green) show multiple Gal3+ and Ki67+ cells at day 8. **E** Quantification of the number of Gal3+ cells. **F** Colocalisation with Ki67 reveals that almost all proliferating cells are also Gal3 + . **G** Representative light microscopy super-resolution images show an accumulation of lipid droplets (Nile Red=red) on day 8. **H** Quantification of increased lipid droplet number after injury on day 8. **I** Representative TEM images highlight the close proximity of lipid droplets (red) to mitochondria (green) after injury. **A**–**I** *n* = 3–4 independent cultures from passaged cells, (**B**,** E**, **F**, **H**) unpaired two-tailed Student’s *t* test, (**C**) two-way ANOVA followed by Tukey’s post-hoc test, *p < 0.05, **p < 0.01, ***p < 0.001, ****p < 0.0001. The bar graphs show mean ± SD.

Astrocytes are known to contribute to ECM remodelling and so-called ‘glial scar’ formation following injury [42, 43]. Wisteria floribunda lectin binds to the glycosaminoglycan side chains of ECM proteins and is commonly used to visualise chondroitin sulphate proteoglycans (CSPGs), which are the most abundant components of ECM remodelling following injury [44]. WFL staining on day 8 revealed a pronounced increase in fluorescence intensity in injured astrocytes compared with untreated controls (*p* = 0.0077) (Fig. 2A, B). In addition, qPCR analysis of two ECM remodelling-associated genes, encoding for Fibronectin [45] and Biglycan [46], showed a non-significant increase in expression over time in injured cultures relative to controls (Fig. 2C).

Another analysed readout was the expression of Galectin-3, an emerging marker of astrocyte reactivity [47]. On day 8, injured samples exhibited more than a three-fold increase in the number of Galectin-3-positive astrocytes compared with untreated controls (*p* = 0.0021) (Fig. 2D, E). The majority of Galectin-3-positive cells were also positive for the proliferation marker Ki67, as indicated by the arrow. Accordingly, injured astrocytes displayed almost a ten-fold increase in Galectin-3/Ki67 double-positive cells on day 8 (*p* = 0.0083) (Fig. 2F).

A further well-described stress response of astrocytes to injury is the formation of lipid droplets [48]. Super-resolution light microscopy revealed a six-fold increase in the number of lipid droplets in injured astrocytes compared with untreated controls (*p* < 0.0001) (Fig. 2G, H). Furthermore, lipid droplets (pseudo-coloured in red) appeared to closely colocalise with mitochondria (pseudo-coloured in green) (Fig. 2I).

Taken together, these results highlight the suitability of the in vitro rmTBI model to induce a distinctive astrocytic phenotype and to recapitulate multiple hallmarks of astrocytic injury that are well established in in vivo studies.

### Repeated mild injury induces mitochondrial stress and time-dependent metabolic reprogramming in human astrocytes rather than acute inflammation

**Fig. 3:**
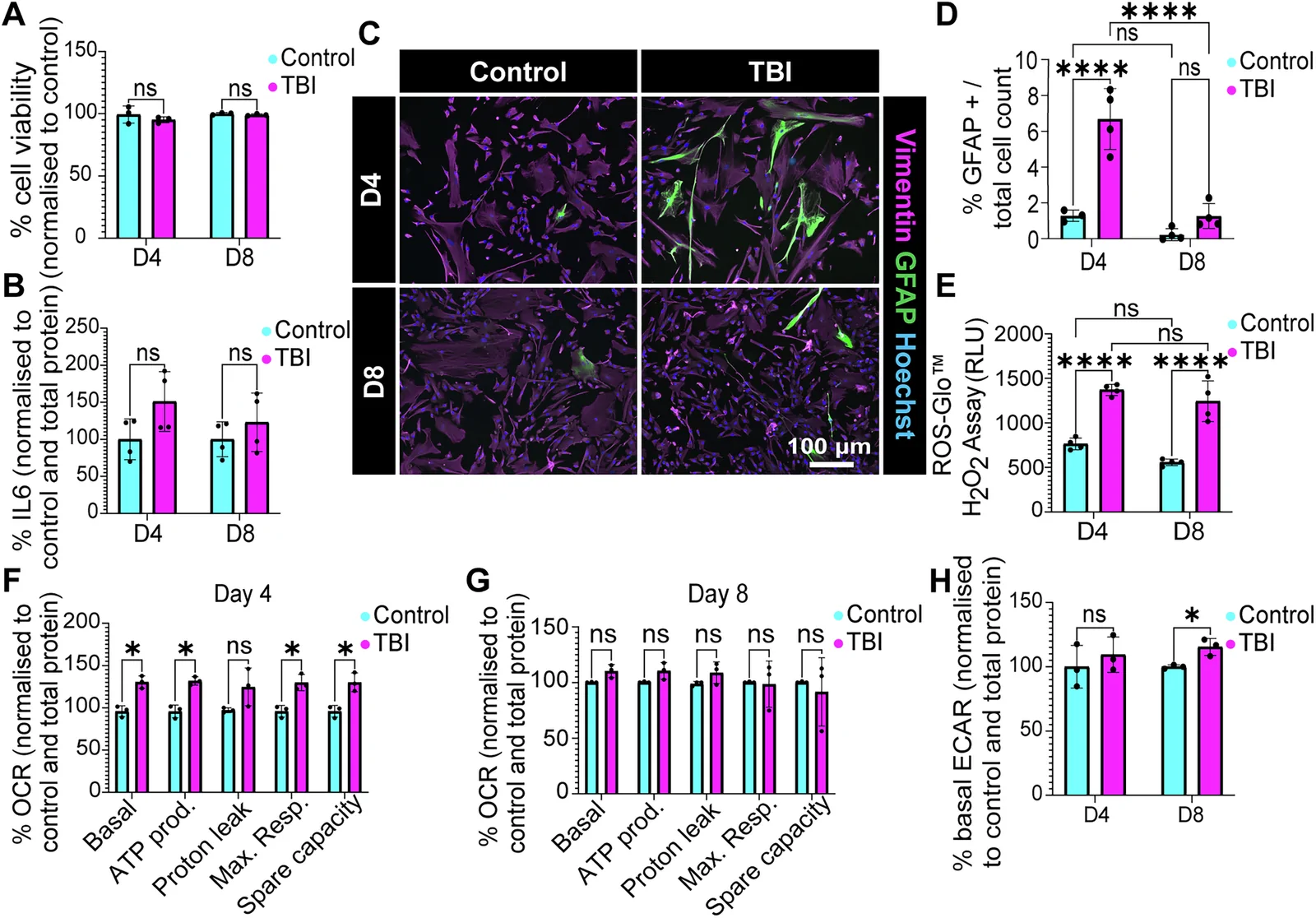
Repeated mild TBI induces metabolic reprogramming and increased oxidative stress in astrocytes. **A** Human primary astrocytes do not display a reduced cell viability or **B** increased levels of the proinflammatory cytokine IL-6 after injury compared to untreated controls. **C** Representative images of immunofluorescence for Vimentin (magenta) and GFAP (green) show a higher number of GFAP+ cells at day 4 compared to day 8. **D** Quantification of the number of GFAP+ cells. **E** Repeated mild TBI induces an increase in reactive oxygen species at day 4 and day 8 in injured astrocytes. **F** Seahorse analyser shows an increase in the OCR on day 4 (**G**) which returns to control levels on day 8. **H** The basal ECAR remains comparable to control levels on day 4 but starts to increase significantly towards day 8. **A**–**H** *n* = 3–4 independent cultures from passaged cells from a single donor, (**A**, **B**, **F**, **G**, **H**) unpaired two-tailed Student’s *t* test, corrected for multiple comparison using the Holm-Šídák method, (**D**, **E**) two-way ANOVA followed by Tukey’s post-hoc test, *p < 0.05, **p < 0.01, ***p < 0.001, ****p < 0.0001. The bar graphs show mean ± SD.

To explore functional changes in human astrocytes, human foetal primary astrocytes isolated from the cortex were used. Similar to observations in immortalised human astrocytes, repeated mild injury did not result in a significant change in cell viability in primary astrocytes compared with untreated controls (Fig. 3A). Interestingly, ELISA analysis of the conditioned medium revealed no significant difference in the concentration of the pro-inflammatory cytokine interleukin-6 (IL-6) following injury (Fig. 3B). In addition, levels of interleukin-1 beta (IL-1β) and tumour necrosis factor alpha (TNFα) in the conditioned medium were below the detection limit in both injured and control conditions (data not shown).

Despite no detected increase in the measured proinflammatory cytokines, primary astrocytes responded to injury with a five-fold increase in the number of glial fibrillary acidic protein (GFAP)-positive cells on day 4 (*p* < 0.0001). This initial increase in GFAP expression in injured cultures declined over time and returned to levels comparable to untreated controls by day 8 (Fig. 3C, D).

Astrocytes are also known contributors to the production of reactive oxygen species (ROS) [49]. Consistent with this, injured astrocytes exhibited a two-fold increase in ROS production on day 4 (*p* < 0.0001). In contrast to the transient increase in GFAP expression, elevated ROS release persisted through day 8 (*p* < 0.0001) (Fig. 3E).

Given this sustained ROS production, mitochondrial function in astrocytes was further investigated using a Seahorse extracellular flux analyser. Basal oxygen consumption rate (OCR) was increased by approximately 30% in injured astrocytes on day 4 compared with untreated controls (*p* = 0.0146). In addition, ATP production (*p* = 0.0115), maximal respiration (*p* = 0.0231) and spare respiratory capacity (*p* = 0.0231) were all equally upregulated at this time point (Fig. 3F). By day 8, this increase in mitochondrial activity was no longer evident, with maximal respiration and spare capacity showing no detectable difference between injured and control cultures (Fig. 3G).

These time-dependent changes in mitochondrial function were also reflected in changes in basal extracellular acidification rate (ECAR). While no significant differences were detected between injured and control cultures on day 4, basal ECAR was significantly increased in injured astrocytes by day 8 (*p* = 0.0329) (Fig. 3H).

Together, these findings indicate that astrocytes underwent a defined and time-dependent phenotypic shift following repeated mild injury, characterised by multiple functional alterations. Notably, these changes predominantly involve metabolic adaptations rather than a classical inflammatory response.

### Human astrocytes exhibit delayed swelling after repeated mild injury

Following TBI, the formation of cerebral oedema is one of the major clinical risks and remains difficult to manage. In astrocytes metabolic alterations may also contribute substantially to tissue swelling by regulating the expression of water channels, including AQP4 [20, 50]. To better understand the contribution of astrocytes to oedema formation, cell volume was analysed using live-cell imaging to exclude fixation-associated artefacts.

**Fig. 4:**
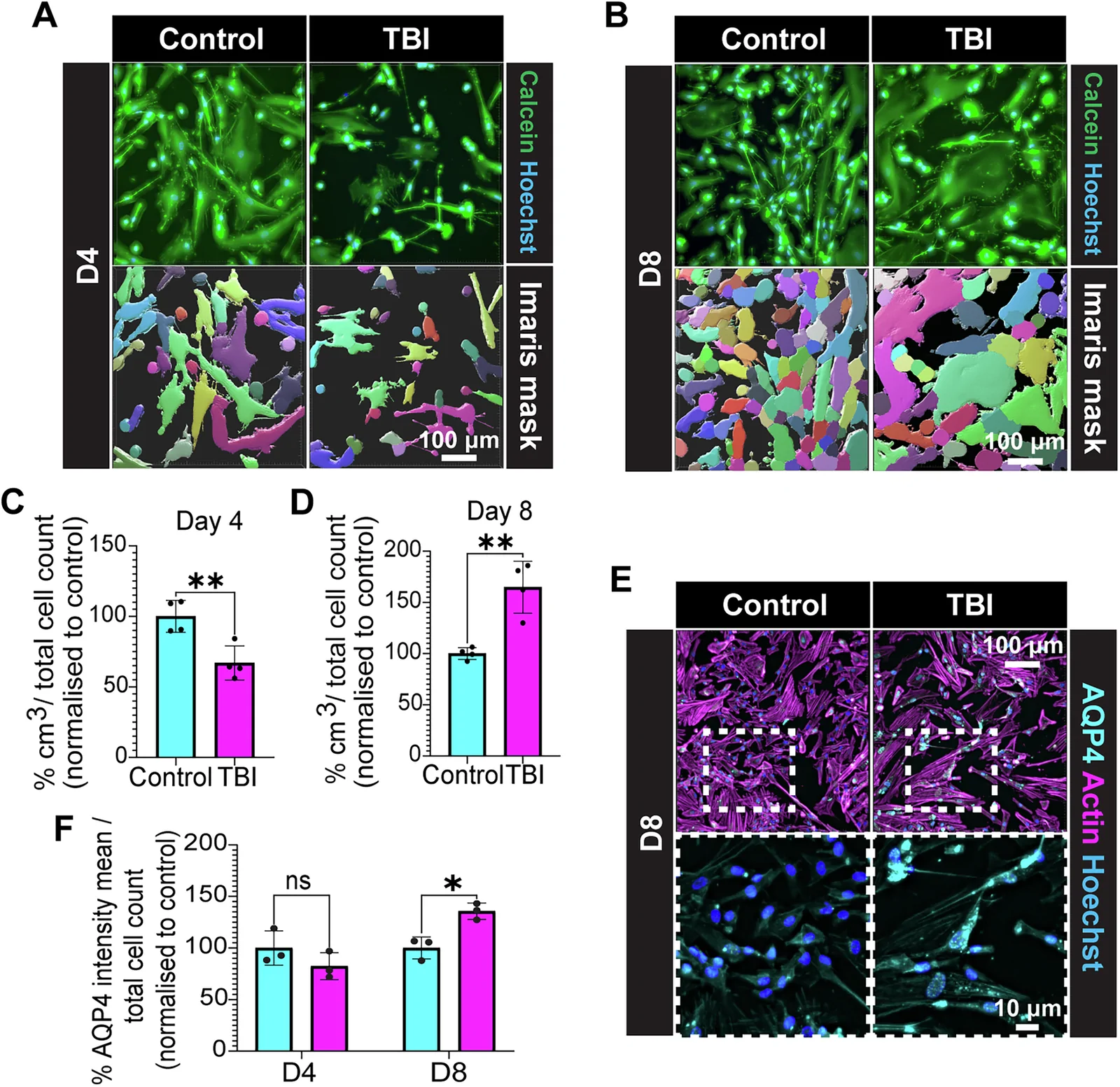
Biphasic astrocyte volume changes and delayed AQP4 upregulation following repeated mild TBI. **A** Representative live-cell images show a reduction in cell volume (Calcein AM=green) following injury on day 4. **B** In contrast on day 8 astrocytes display an increased volume following injury compared to control samples. **C** Quantification of cell volume for day 4 and **D** day 8. **E** Representative images of immunofluorescence for AQP-4 (cyan) and Actin (magenta) show an increase in AQP4 signal on day 8 in injured astrocytes. **F** Quantification of AQP4 immunofluorescence for day 4 and day 8. **A**–**E** *n* = 3-4 independent cultures from passaged cells from a single donor, (**C**, **D**) unpaired two-tailed Student’s t-test, **F** unpaired two-tailed Student’s t-test, corrected for multiple comparisons using the Holm-Šídák method, *p < 0.05, **p < 0.01, ***p < 0.001, ****p < 0.0001. The bar graphs show mean ± SD.

Unexpectedly, on day 4, the cell volume of injured astrocytes was not increased but instead was 25% reduced compared with untreated controls (*p* = 0.0071) (Fig. 4A, C). In contrast, by day 8 the volume of injured astrocytes was doubled relative to controls (*p* = 0.0025) (Fig. 4B, D). Consistent with these delayed volumetric changes, AQP4 expression was not significantly altered on day 4 (Fig. 4F) (representative images, Fig. 2, Supporting Information). However, by day 8, there was a 25% increase in AQP4 expression in injured astrocytes (*p* = 0.0097) (Fig. 4E, F).

Therefore, these results reveal that repeated mild injury induces a biphasic astrocytic volume response, characterised by an initial reduction in cell volume followed by delayed swelling associated with increased AQP4 abundance.

### Repeated mild injury leads to increased release of clusterin/APOJ and diacylglycerols from human astrocytes

Astrocytes play a central role in maintaining brain homeostasis, in particular by supporting an optimal environment for neuronal function. Previous studies have shown that reactive astrocytes can induce neurotoxicity through the release of lipoprotein particles enriched in saturated lipids [26].

**Fig. 5:**
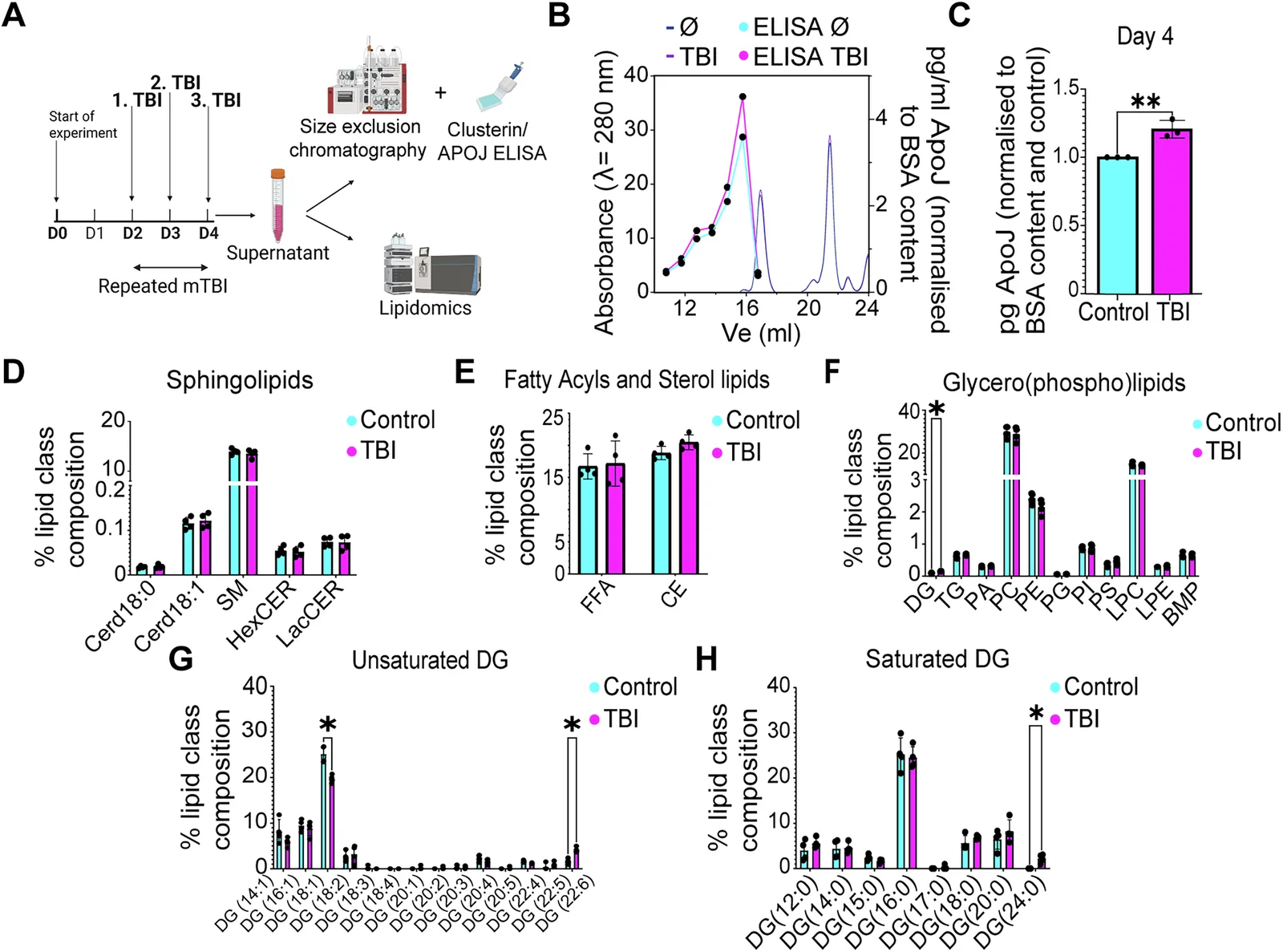
Injured astrocytes secrete clusterin/APOJ and diacylglycerols. **A** Schematic of conditioned medium analysis. Medium on day 4 was examined for lipoproteins using SEC and subsequent ELISA and lipid composition was detected by lipidomics. **B** SEC chromatogram of conditioned medium overlaid with ELISA-based clusterin/APOJ quantification reveals higher secretion of clusterin/APOJ from injured astrocytes. **C** Quantification of total clusterin/APOJ concentration in the medium by ELISA on day 4. Lipidomics analysis of astrocyte-conditioned medium at day 4 reveals no significant differences between injured and control groups in sphingolipids (**D**) or fatty acyl and sterol lipids (**E**). **F** Lipid class composition of glycerophospholipids shows a significant upregulation of diacylglycerols (DG) in conditioned medium from injured astrocytes compared with controls. Lipid class concentrations of unsaturated DGs (**G**) and saturated DGs (**H**) are shown. **B**–**G** *n* = 3–4 independent cultures from passaged cells from a single donor, **C** unpaired two-tailed Student’s *t* test, (**D**, **E**, **F**, **G**) unpaired two-tailed Student’s *t* test, corrected for multiple comparisons using the Holm-Šídák method, *p < 0.05, **p < 0.01, ***p < 0.001, ****p < 0.0001. The bar graphs show mean ± SD.

To further understand these neurotoxic processes and the factors involved, the conditioned medium from injured astrocytes was analysed (Fig. 5A). As an initial readout for potential changes in lipoparticle secretion, the abundance of clusterin/APOJ was assessed. Conditioned medium was fractionated by size-exclusion high-performance liquid chromatography (SEC) to isolate HDL-like particle fractions. ELISA was then used to quantify clusterin/APOJ levels in the relevant fractions from injured astrocytes and untreated controls. In the chromatogram a characteristic monomer-dimer peak for bovine serum albumin was observed. The elution profile of clusterin/APOJ showed a main peak in fraction 11 (hydrodynamic diameter ≈ 9.6-12.2 nm) and a smaller shoulder at lower elution volumes (hydrodynamic diameter > 17 nm) (Fig. 5B). Consistent with the hypothesis that injured astrocytes release potentially neurotoxic substances, clusterin/APOJ levels were increased by approximately 25% in conditioned medium from injured astrocytes on day 4 compared to controls (*p* = 0.0055) (Fig. 5C).

To determine which lipid species were associated with these lipoprotein particles, shotgun lipidomics analysis across 17 lipid classes was performed. No significant differences were detected in the abundance of sphingolipids (Fig. 5D), fatty acids or sterol lipids in conditioned medium from injured versus control astrocytes (Fig. 5E). In contrast, within the glycerophospholipids group, diacylglycerols (DG) showed a significant change in abundance (Fig. 5F), with conditioned medium from injured astrocytes displaying higher DG levels compared to controls (*p* = 0.0121). Notably, cholesterol levels in the conditioned medium, while not being significant, were increased as well (*p* = 0.0750).

Analysis of individual DG species revealed that the unsaturated DG(18:1) was reduced by 25% following injury (*p* = 0.0221), whereas the polyunsaturated DG(22:6) was increased by more than two-fold (*p* = 0.0157) (Fig. 5G). Among saturated DG species, only DG(24:0) was significantly altered (*p* = 0.0291) (Fig. 5H). Interestingly, DG(24:0) was detected exclusively in conditioned medium from injured astrocytes at around 3% and was absent from all control samples.

In light of previous findings implicating saturated lipids as particularly detrimental, the increased appearance of DGs in injured astrocyte-conditioned medium identifies this lipid class as a potentially relevant factor in astrocyte-mediated neurotoxicity.

### Injured astrocytes induce apoptotic effects in cortical neurons via diacylglycerol release and subsequent PKCδ activation

**Fig. 6:**
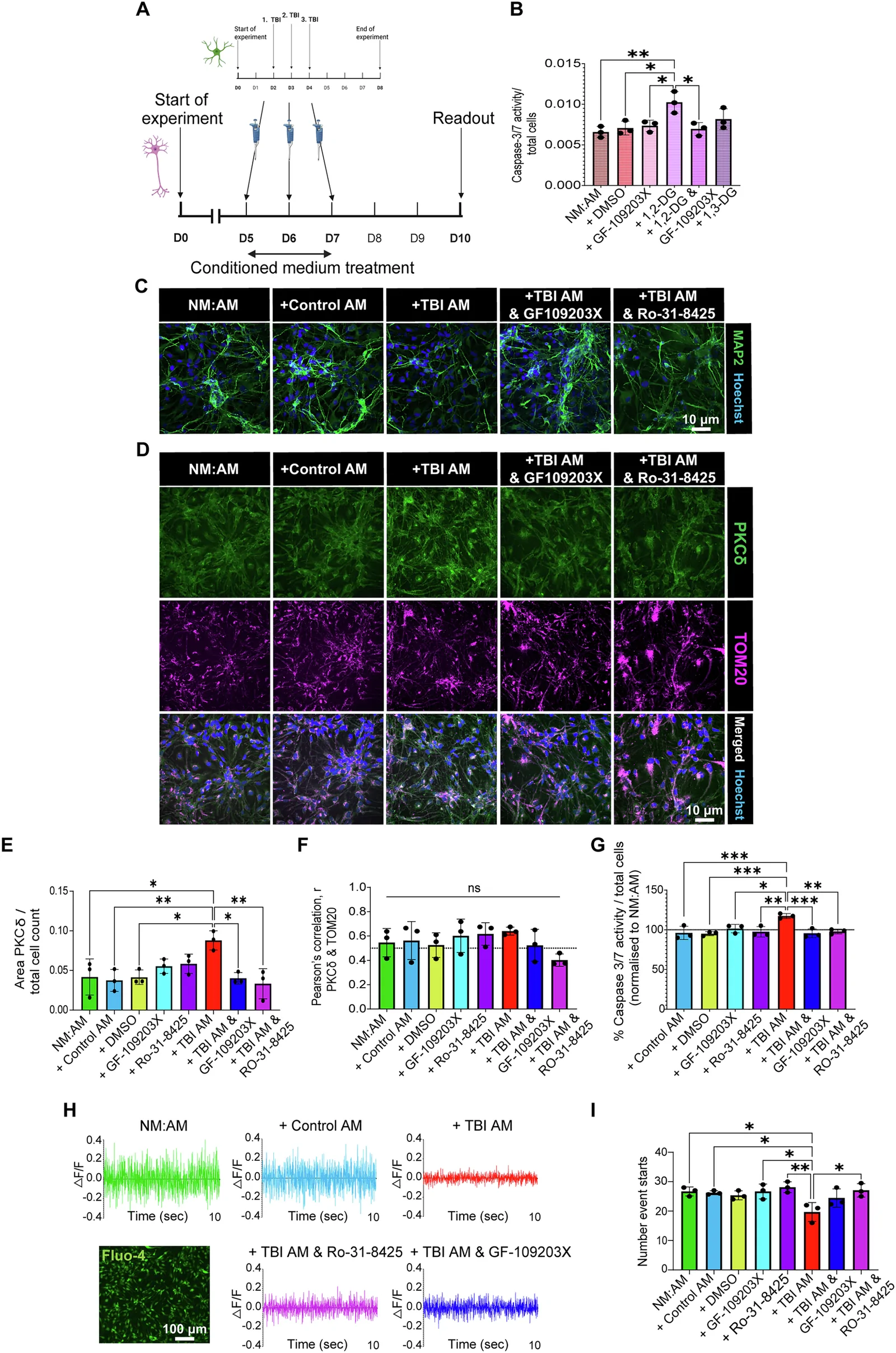
Injured astrocytes induce PKCδ-dependent neuronal apoptosis and dysfunction in human cortical neurons. **A** Schematic of conditioned medium treatment of primary human neurons. **B** Quantification of apoptosis effector caspases-3 and -7 shows an increase in apoptosis in neurons treated with 1,2-DG compared to the 1,3-isoform and controls. Addition of the PKC inhibitor GF-109203X reduces activity levels back to baseline; data points describe technical replicates of one donor. **C** Representative images of immunofluorescence for MAP2 (green) show reduced neuronal networks for cultures incubated with conditioned medium from injured astrocytes. **D** Representative images of immunofluorescence for PKCδ (green), mitochondria (TOM20) (magenta) and merged images. **E** Quantification of PKCδ positive-area normalised to cell count shows an increase in PKCδ in neurons treated with conditioned medium from injured astrocytes compared to conditioned medium from control astrocytes or fresh astrocyte/neuron medium. Addition of the PKC inhibitors GF-109203X and Ro-31-8425 reduces PKCδ levels back to baseline. **F** Pearson’s correlation (r) between PKCδ and mitochondria location reveals a robust colocalisation between PKCδ and mitochondria especially in samples treated with conditioned medium from injured astrocytes. **G** Quantification of apoptosis effector caspases-3 and -7 normalised to total cells shows an increase in apoptosis in neurons treated with conditioned medium from injured astrocytes compared to controls. Addition of the PKC inhibitors GF-109203X and Ro-31-8425 reduces apoptosis levels back to baseline. **H** Representative signals obtained from real-time bulk calcium imaging and Fluo-4 loading control (green). **I** Calcium activity analysis displays a reduction in event numbers for cultures treated with conditioned medium from injured astrocytes compared to controls and is rescued by the addition of the PKC inhibitors GF-109203X or Ro-31-8425. Representative immunofluorescence images and signal of calcium imaging of DMSO, GF-109203X and Ro-31-8425 can be found in Supporting Information Figs. 4,5 respectively. **B** *n* = 1 independent culture from single neuronal donor, (**C**–**I**) *n* = 3 independent cultures from three donors, each treated with conditioned medium pooled from three independent astrocyte cultures from three donors, (**D**–**F**, **H**) one-way ANOVA followed by Tukey’s post hoc test, *p < 0.05, **p < 0.01, ***p < 0.001, ****p < 0.0001. The bar graphs show mean ± SD.

To further investigate astrocyte-neuron crosstalk, we tested the hypothesis that conditioned medium derived from injured astrocytes exerts direct neurotoxic effects. Primary human foetal cortical neurons were exposed for three consecutive days to conditioned medium from injured astrocytes (TBI AM), conditioned medium from untreated astrocytes (Control AM) or fresh astrocyte medium (NM:AM) (Fig. 6A). To minimise donor-specific variability, conditioned medium from three independent donors was pooled using a combined approach. The ratio of neuronal medium to astrocyte-conditioned medium was maintained at 1:1 across all conditions as a standard protocol. Control experiments confirmed that the mixed medium itself did not affect neuronal viability (Fig. 3, Supporting Information).

Given that DGs are established activators of protein kinase C (PKC) and PKCδ is a well-described pro-apoptotic effector, we first used the broad-spectrum PKC inhibitors GF-109203X (0.05 µM) and Ro-31-8425 (0.5 µM) to determine whether PKC signalling was required and then examined PKCδ as the candidate downstream isoform [51, 52].

PKCδ is known to induce apoptosis through activation of caspase-3 [52, 53]. Therefore, we initially sought to clarify the involvement of DGs in caspase-3/7 activation by treating cultured cortical neurons with an individual DG type. This reductionist approach allowed us to assess the direct contribution of specific DGs before progressing to more complex conditions. The 1,2-DG isoform represents the physiologically relevant configuration, whereas 1,3-DG served as an isomeric control. Quantification of caspase-3/7 revealed a 2-fold increase in apoptotic activity in neurons treated with 1,2-DG compared with controls (*p* = 0.0064) (Fig. 6B). Caspase-3/7 activity was reduced to baseline levels upon co-treatment with the PKC inhibitor GF-109203X. Subsequently, we expanded our analysis to include multiple donors and the complete conditioned medium, enabling a more comprehensive evaluation of combined effects and their impact on neuronal function.

Neurons from three different donors treated with conditioned medium from injured astrocytes exhibited a less complex morphology when stained for MAP2, with a marked reduction in network-like structures compared to controls. This effect was reversed upon co-treatment with PKC inhibitors (Fig. 6C). In addition, conditioned medium from injured astrocytes led to increased PKCδ levels by 50% in cortical neurons relative to controls (*p* = 0.0176), as determined by immunocytochemistry (Fig. 6D, E). Moreover, the PKCδ signal showed strong colocalisation with mitochondria (TOM20) (r mean = 0.64), suggesting not only increased accumulation but also mitochondrial translocation, indicative of PKCδ activation and supporting its role in the observed mechanism (Fig. 6F). These effects were attenuated to control levels following treatment with GF-109203X and Ro-31-8425.

Next, the activity of effector caspases 3 and 7 after full conditioned medium treatment was also measured in cortical neurons. Neurons exposed to conditioned medium from injured astrocytes showed a small but robust increase in apoptotic activity compared to untreated controls (*p* = 0.0040) (Fig. 6G). This increase was abolished upon co-treatment with PKC inhibitors, restoring apoptosis levels to baseline.

To evaluate the functional consequences of these changes, neuronal calcium activity was assessed in real-time. This approach measures bulk calcium signalling across the culture, providing an indication of overall network functionality (Fig. 6H, I). While control neurons displayed robust activity, exposure to conditioned medium from injured astrocytes resulted in a marked reduction in event starts by around 25% (*p* = 0.0246). Inhibition of PKC largely restored neuronal activity.

These findings support a DG-responsive, PKC-dependent mechanism of astrocyte-associated neurotoxicity, with PKCδ emerging as the most likely downstream effector. Cortical neurons exposed to conditioned medium from repeatedly injured astrocytes showed increased PKCδ accumulation, altered morphology, elevated caspase 3/7 activity and impaired functional output.

## Discussion

TBI remains a major cause of long-term neurological disability. rmTBI is often conceptualised as a transient mechanical insult, yet the cellular mechanisms that convert repeated injury into delayed neurodegeneration remain poorly defined. A central limitation has been the lack of human systems that can isolate cell-intrinsic responses to repeated mechanical stress while retaining experimental control. Here, we establish a modular human TBI-in-a-dish platform and show that repeated mild injury induces a defined astrocytic state marked by metabolic remodelling and release of DG-enriched conditioned medium that is sufficient to activate PKC signalling and impair cortical neurons. This response is not accompanied by astrocyte death or a classical cytokine profile but instead reflects sustained stress-associated lipid remodelling linked to PKCδ-dependent neuronal dysfunction. These findings identify astrocytes as active mediators of delayed neurotoxicity after rmTBI, rather than passive responders to injury.

A key strength of this model is that it preserves astrocyte viability while eliciting a defined and time-resolved injury response. This is important because repeated mild TBI is clinically common, yet its long-term consequences are often underestimated [54, 55]. In our system, repeated injury was sufficient to induce ECM remodeling [56], Galectin-3 expression associated with proliferative reactivity [57] and lipid droplet accumulation, all of which are consistent with established astrocytic injury responses in vivo. The model therefore recapitulates multiple hallmarks of reactive astrogliosis while remaining sufficiently reductionist to resolve astrocyte-intrinsic mechanisms under controlled conditions. The accumulation of lipid droplets is particularly interesting, as this phenotype is increasingly recognised as a stress-associated astrocytic response with relevance to both injury and neurodegeneration [48].

The astrocytic response was not dominated by a classical cytokine programme. Although astrocytes are well-known contributors to neuroinflammation [19], we did not detect a significant increase in the inflammatory cytokines analysed under these conditions. Instead, repeated mild injury induced sustained oxidative stress and time-dependent metabolic reprogramming. The early increase in mitochondrial activity is consistent with an adaptive response to injury, whereas the later shift towards glycolysis and reduced respiratory reserve suggests progressive metabolic failure. As reviewed by Tiwari and Simons [58], reactive astrocytes tend to favour glycolysis, whereas astrocytes engaged in resolution rely more on oxidative phosphorylation. This temporal pattern argues for a transition from compensation to dysfunction, rather than a uniform inflammatory activation state.

The biphasic change in cell volume further supports this interpretation. Oedema formation is a common consequence of CNS injuries, with limited treatment options and a strong association with increased patient mortality [59]. Astrocytes are widely reported to take up interstitial fluid after injury, leading to cell swelling and a substantial contribution to oedema development [60]. In contrast to this prevailing view and immediate swelling, injured astrocytes showed an early reduction in volume followed by delayed enlargement accompanied by increased AQP4 abundance. Previous studies have shown that AQP4-deficient mice display improved survival following acute water intoxication compared to wild-type animals [61] and dysregulation of AQP4 has been reported in postmortem brain tissue from TBI patients [62]. While rapid astrocyte swelling within minutes of acute osmotic or ischemic stress has been demonstrated in cortical slices [63], other studies highlight a more context-dependent astrocytic response, in line with our observations [64, 65]. This is important because it suggests that astrocytic water handling after repeated mild injury is dynamic and delayed, rather than simply reflecting an acute oedematous response. AQP4 upregulation may therefore represent part of a later maladaptive state that contributes to tissue vulnerability. In this context, the volume phenotype is best interpreted as part of a broader injury programme in which astrocytes evolve over time from an initially compensated state towards delayed dysfunction.

The most consequential finding is that astrocyte-conditioned medium from repeatedly injured cultures was sufficient to drive neuronal dysfunction. The neurotoxic potential of reactive astrocytes has been reported previously; for example, Guttenplan et al. showed that cytokine-treated rat astrocytes release HDL-like, saturated lipids packaged in apolipoproteins that induce cell death [26]. Consistent with these findings, we detected elevated levels of apolipoprotein J (Clusterin) in the conditioned medium of human astrocytes following rmTBI. Clusterin accumulation has been reported in postmortem tissue from patients with Alzheimer’s disease [66] and Clusterin has been described as a chaperone modulating β-amyloid toxicity [67]. However, Guttenplan et al. further showed that the lipoprotein carriers themselves are not directly neurotoxic; rather, toxicity is mediated by the lipid cargo.

Therefore, we performed shotgun lipidomics and found the neurotoxic output to be accompanied by selective enrichment of diacylglycerols, pointing to a lipid-based secretory programme. Earlier lipidomics studies in experimental and clinical TBI have identified persistent alterations in glycerophospholipids, sphingolipids and polyunsaturated fatty acids, changes that correlate with axonal injury and clinical outcome [27–29]. Moreover, DG accumulation has previously been reported in serum [68] and cortical tissue [69] after impact injury in rodent in vivo models. DGs have also been implicated in the regulation of systemic inflammation, aligning with the non-inflammatory phenotype we observed in injured astrocytes [70]. Beyond this, diacylglycerols are not merely by-products of membrane damage; they are canonical second messengers capable of activating PKC-family signaling [71]. Alterations in PKC signalling have been described in rodent models of TBI previously [72]. In neurons exposed to conditioned medium from injured astrocytes, we observed PKCδ accumulation and mitochondrial association, indicative of its active state. PKCδ belongs to the novel PKC subfamily, is calcium-independent and is primarily activated by DGs. Importantly, PKCδ is widely recognised as a key pro-apoptotic effector, mediating DG-induced activation of caspase-3 cleavage [52, 53].

Consistent with this, neurons exhibited increased caspase 3/7 activity, together with impaired calcium signalling, all of which were attenuated by PKC inhibition. Previous studies have shown that suppression of PKCδ protects rodent dopaminergic neurons from apoptosis [73] and reduces microglia-driven neuroinflammation [74]. These data support a model in which repeated mild mechanical injury is converted by astrocytes into a DG-enriched lipid signal associated with neuronal injury through a PKC-dependent pathway, with PKCδ emerging as the strongest downstream effector.

This mechanism is notable for two reasons. First, it shifts the focus from broad astrocyte reactivity to a more specific biochemical route linking repeated injury to neuronal damage. Second, it suggests that delayed neurotoxicity after rmTBI may emerge from persistent lipid metabolic reprogramming rather than from an immediate cytokine-driven inflammatory response. That distinction matters because it defines a mechanistically tractable window between injury and degeneration. In this context, astrocytes appear to exhibit a form of ‘injury memory’: repeated sublethal insult is not simply resolved once the acute response subsides, but leaves a persistent imprint on astrocyte metabolism, secretory output and water homeostasis.

Several limitations should be noted. The model uses cells of foetal origin, which may behave differently from more mature or aged cells. It also isolates astrocyte-intrinsic responses and therefore does not capture the full multicellular and vascular complexity of TBI, including microglia, endothelial cells, pericytes and infiltrating immune cells. Nor does it fully reconstruct the biomechanics of in vivo head injury. However, this reductionism is also its advantage: it allows repeated mild injury to be studied in a controlled human system, making it possible to resolve delayed astrocytic programmes that are difficult to separate in animal models. The platform is therefore best viewed not as a replacement for in vivo systems, but as a complementary model that defines human cell-biological mechanisms upstream of more complex tissue responses. Moreover, it is important to recognise that DG release and subsequent PKCδ activation are unlikely to represent the sole mechanism by which injured astrocytes impair neuronal function. While this study centres on PKCδ as a DG-responsive, apoptosis-associated isoform, contributions from other PKC family members cannot be excluded. In addition, astrocyte-derived factors such as ROS may further modulate neuronal viability and function. Future work will be required to dissect the relative contributions and potential interplay of these pathways.

In summary, this study establishes a modular and scalable human TBI-in-a-dish model and identifies repeated mild injury as a driver of persistent astrocyte reprogramming rather than a transient stress response. Across complementary readouts, rmTBI induced a delayed reactive state characterised by cytoskeletal remodelling, oxidative and metabolic stress, lipid droplet accumulation and the release of a distinct bioactive lipid signature into the conditioned medium. Importantly, these astrocyte-derived factors were sufficient to trigger a DG-enriched lipid signal linked to neuronal injury processes and this toxicity was attenuated by PKC inhibition, identifying a mechanistically tractable pathway connecting repeated mechanical insult to delayed neurotoxicity. Together, these findings support a model in which human astrocytes retain a form of ‘injury memory’ that persists beyond the initial impact and is translated into a DG-enriched lipid signal that may contribute to neuronal injury. By defining the DG-PKCδ axis as a candidate effector of this response, the work reframes rmTBI as a model not only of acute mechanical injury but also of delayed glial-driven degeneration and highlights lipid signaling as a previously underappreciated and potentially targetable pathway for preventing the long-term consequences of repeated mild brain injury.

## Supplementary information

Supportive information

## Data Availability

The data that support the findings of this study are available from the corresponding author upon reasonable request.
