# Supplementary material for: From injury to degeneration: delayed lipid-mediated neurotoxicity in a human astrocyte traumatic brain injury in-a-dish model
Source: Cell Death Dis. 2026 Aug 24;17(1):911. doi: 10.1038/s41419-026-09217-8 (PMC13713977; doi:10.1038/s41419-026-09217-8)
Supplement: Supplementary file 1 [file 41419_2026_9217_MOESM1_ESM.docx]

**Supportive Information**

**From injury to degeneration: delayed lipid-mediated neurotoxicity in a human astrocyte traumatic brain injury in-a-dish model**

Luise Schlotterose^1,2,3,4^, Michel A. Struwe^5^, Axel J. Scheidig^5^, Niek Blomberg^6^, Martin Giera^6^, Kirsten Hattermann^7^ & Mootaz M. Salman^1,2,3,4^

*^1^Department of Physiology, Anatomy, and Genetics, University of Oxford, Oxford OX1 3PT, UK.*

*^2^British Heart Foundation (BHF) - UK Dementia Research Institute (UK DRI) Centre for Vascular Dementia Research (CVDR) at the University of Oxford, Oxford, UK.*

*^3^Kavli Institute for NanoScience Discovery, University of Oxford, Oxford, UK.*

*^4^British Heart Foundation (BHF) Oxford Centre of Research Excellence, University of Oxford, Oxford, UK.*

*^5^Department for Structural Biology, Kiel University, Kiel, Germany*

*^6^Leiden University Medical Center, Center for Proteomics and Metabolomics, Leiden, The Netherlands*

*^7^Institute of Anatomy, Kiel University, Kiel, Germany*

***Supportive information figure 1****: Secondary antibody controls. To test secondary antibody specificity primary antibodies were omitted. (A) Galectin-3 & Ki67, (B) Vimentin & GFAP (C) AQP4 (D) MAP2. To test Avidin specificity initial Lectin staining was omitted (E) WFL.*

***Supportive information figure 2****: Representative images of AQP4 staining on day 4 (AQP4 cyan), Actin (magenta), nuclei (blue).*

***Supportive information figure 3****: Human primary cortical neurons show no decrease in cell viability when grown in 1:1 NM:AM medium.*

***Supportive information figure 4****: Representative images of + DMSO, +GF109203X, +Ro-31-8425 (A) MAP2 (green), (B) PKC delta (green), TOM20 (magenta), nuclei (blue).*

***Supportive information figure 5****: Representative calcium traces of + DMSO, +GF109203X, +Ro-31-8425*
